# Aseptic Orbital Cellulitis as a Complication of Suprachoroidal Hemorrhage

**DOI:** 10.7759/cureus.35528

**Published:** 2023-02-27

**Authors:** Maja Magazin, Venkatkrish M Kasetty, Dilip A Thomas

**Affiliations:** 1 Ophthalmology, Augusta University Medical College of Georgia, Augusta, USA; 2 Ophthalmology, Henry Ford Health System, Detroit, USA

**Keywords:** panophthalmitis, intraocular hemorrhage, spontaneous suprachoroidal hemorrhage, sterile orbital inflammation, aseptic orbital cellulitis

## Abstract

Suprachoroidal hemorrhage is a rare and potentially devastating clinical entity seen in individuals on anticoagulation presenting with severe unilateral eye pain, sudden vision loss, and elevated intraocular pressures. Herein, we report the first case of aseptic orbital cellulitis caused by recurrent spontaneous suprachoroidal hemorrhage. This case highlights an example of non-infectious orbital cellulitis arising from choroidal pathology in the setting of uncontrolled intraocular pressures and recurrent intraocular bleeding. Surgical intervention with blood drainage should be considered to prevent complications and preserve the globe.

## Introduction

Suprachoroidal hemorrhage (SCH) is a rare, sight-threatening entity that may occur spontaneously, following intraocular surgery, or in the setting of trauma [1]. Clinically, SCH may present with severe unilateral eye pain, sudden severe vision loss, and elevated intraocular pressures. Typically, the visual prognosis is guarded, especially when associated with a retinal detachment, choroidal apposition, or secondary angle closure glaucoma [2]. Early medical and surgical interventions are crucial in preserving the globe, as globe perforations are a devastating complication [1]. Herein, we present an unusual case of aseptic orbital cellulitis as a complication of a non-resolving, recurrent, SCH. This article was previously presented as a meeting abstract at the Women in Ophthalmology Summer Symposium on August 20, 2021.

## Case presentation

A 59-year-old female with a history of atrial fibrillation on warfarin, decompensated cirrhosis, cardiomyopathy, diabetes, and a history of chronic angle closure glaucoma presented with five days of severe pain and decreased vision in the right eye. The international normalized ratio (INR) was supratherapeutic at 5. Visual acuity (VA) was no light perception (NLP) in the right eye and 20/40 in the left. Intraocular pressure (IOP) was 48 and 14 mmHg in her right and left eyes, respectively. The right eye examination was significant for conjunctival injection, corneal edema, and marked shallowing of the anterior chamber, with a hyphema, cell and flare. B-scan ultrasonography revealed appositional (kissing) hemorrhagic choroidal detachment in the right eye. These findings were consistent with a spontaneous SCH. The patient was admitted for pain management and IOP control with low-dose narcotics (oxycodone), aqueous suppressants (brimonidine, timolol, dorzolamide), prednisolone acetate, atropine, and intravenous acetazolamide with subsequent improvement in pain and an IOP of 30. Surgical intervention was discussed with the patient but ultimately deferred given the resolution of pain, the lack of visual potential with NLP vision, and ongoing cardiac complications. The ocular examination remained stable throughout the hospital course, and the INR was stable at 1.8 on discharge after the discontinuation of warfarin.

**Figure 1:**
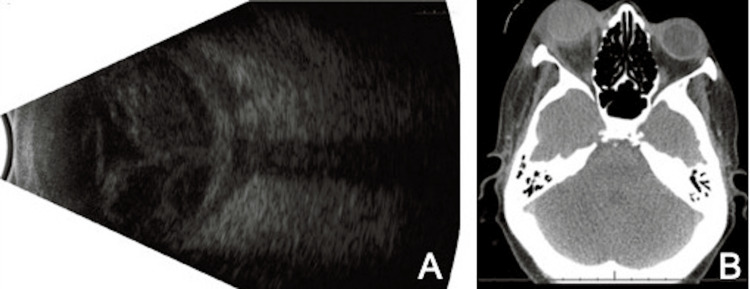
Imaging of Suprachoroidal Hemorrhage A) B-scan ultrasonography of choroidal hemorrhage demonstrating a “T sign” and “kissing choroidals” with associated vitreous hemorrhage. B) Axial CT scan of orbit revealing right peri-global edema and inflammation with diffuse vitreous humor attenuation which in the setting of proptosis, resistance of retropulsion, and new adduction defect raised suspicion for orbital cellulitis. CT: Computed tomography

**Figure 2:**
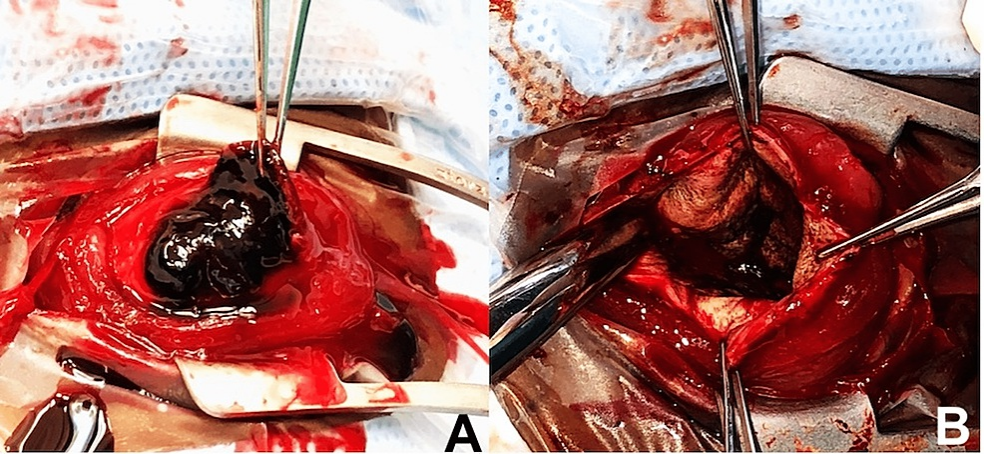
Intraoperative Examination of Globe Inflammation Intraoperative gross examination showing the globe before (A) and after (B) removal of intraocular coagulated blood.

Nine days later, the patient presented with new-onset severe chemosis, proptosis, resistance to retropulsion, and a new adduction defect in the right eye. VA was still NLP, and IOP was elevated at 70. The INR was stable at 1.6. B-scan ultrasonography revealed “kissing choroidals” with associated vitreous hemorrhage (Figure 1A). Although the differential included recurrent intraocular hemorrhage in the setting of known choroidal detachment, the new clinical findings suggested an interval development of an acute intraocular and orbital process. Computed tomography (CT) demonstrated proptosis and inflammatory changes along the posterior sclera with diffuse vitreous humor attenuation of the right eye (Figure 1B), and labs were significant for an interval development of leukocytosis. The patient was admitted to the hospital, was started on broad-spectrum intravenous antibiotics, and subsequently underwent urgent evisceration for concerns of an infectious intraocular process with orbital extension. Intraoperatively, the entire globe was filled with dense coagulated blood (Figure 2). Eye and blood culture were negative, and the patient demonstrated steady clinical improvement. Pathology revealed acute inflammation with coagulated blood and tiny foci of necrosis. The patient had an uncomplicated post-operative course.

## Discussion

SCH is associated with several risk factors including age, anticoagulation, hypertension, diabetes, glaucoma, high myopia, and recent ocular surgery [3,4]. Our patient had several risk factors including a supratherapeutic INR which was likely a result of her cirrhosis and polypharmacy, which included several diuretics, anti-hypertensives, beta-blockers, and calcium channel blockers. As seen in our patient, an acute SCH commonly presents with sudden vision loss and acute angle closure due to the mass effect of extravasated blood narrowing the anterior chamber angle [3,5]. While the main goals of management in the acute phase include vision and globe preservation with pain and IOP control, surgical intervention with blood drainage with sclerotomy or vitrectomy is recommended 10 to 14 days after onset to achieve optimal anatomical and visual outcomes [1,3]. Generally, delayed surgical intervention is recommended to allow for liquefaction of the clot. However, recent studies have demonstrated the use of tissue plasminogen activators to achieve earlier thrombolysis and prevent more serious chorioretinal complications [6]. Although visual outcomes following SCH remain poor with most individuals achieving only hand motion VA despite surgical intervention, recurrent intraocular bleeding may lead to uncontrolled IOP refractory to medical therapy, chorioretinal disruptions, and intraocular inflammation which may lead to intractable pain or globe perforation requiring enucleation or evisceration [1,6].

In our case, surgery was initially deferred after pain and IOP control given multi-organ complications including ongoing cardiac decompensation requiring surgical intervention. Despite normalization of the INR, the patient developed new clinical signs suggestive of orbital inflammation which in combination with imaging findings was concerning for an infectious process. The patient’s immunosuppressive state in the setting of advanced liver cirrhosis and diabetes, and recent invasive cardiological procedures involving a mitral valve repair, left atrial appendage ligation, and implantation of a cardioverter- defibrillator, increased her risk of endogenous endophthalmitis and therefore warranted an urgent evisceration [7]. However, cultures and pathology results were negative for an infectious etiology, and the acute orbital signs were attributed to a recurrent SCH clinically mimicking endophthalmitis with secondary orbital cellulitis. One study reported a choroidal detachment with associated orbital cellulitis with a culture-confirmed infectious etiology [8].

The interval development of an acute sterile orbital process was likely a result of recurrent intraocular bleeding causing recurrent angle closure [9]. We theorize that the sterile orbital inflammation was a result of a malignant inflammatory response caused by high-pressure trans-scleral extravasation of the acute and chronically retained intraocular hemorrhage. This process was supported by the posterior scleral enhancement seen on CT imaging and the “T-sign” on B-scan ultrasonography, indicating fluid behind the posterior sclera (Figure 1). While less aggressive methods such as a posterior sclerotomy or vitrectomy could have been attempted, evisceration was performed due to the poor visual potential and inability to rule out an infectious etiology in a high-risk individual. It is possible that drainage of the choroidal hemorrhage during the first hospitalization may have preserved the globe.

Aseptic orbital cellulitis secondary to retinal or choroidal pathology is rare but has been reported in the setting of intraocular tumors such as retinoblastoma and choroidal melanoma [5]. Orbital inflammation with intraocular malignancy has been attributed to a robust systemic inflammatory response caused by aseptic tissue necrosis in the setting of uncontrolled rapid tumor growth. The case herein highlights an additional mechanism of sterile orbital inflammation caused by intraocular pathology that should be recognized by clinicians as potential complications may impact follow-up care.

## Conclusions

To our knowledge, we report the first case of aseptic orbital cellulitis as a potential complication of a non-resolving SCH. Retained blood products in the setting of uncontrolled IOP and recurrent intraocular bleeding may trigger an extraocular inflammatory response and masquerade as an infectious orbital process, which may pose a diagnostic challenge, especially in immunocompromised individuals. While pain control and pressure control are crucial, early surgical intervention with SCH drainage should be considered to prevent complications and preserve the globe.
